# Concordance between SARS-CoV-2 index individuals and their household contacts on index individual COVID-19 transmission cofactors: a comparison of self-reported and contact-reported information

**DOI:** 10.1186/s12889-024-18371-7

**Published:** 2024-04-02

**Authors:** Angela M. Dahl, Clare E. Brown, Elizabeth R. Brown, Meagan P. O’Brien, Ruanne V. Barnabas

**Affiliations:** 1 Department of Biostatistics, University of Washington, Seattle, WA US; 2 Department of Global Health, University of Washington, Seattle, WA US; 3 Vaccine and Infectious Disease Division and Public Health Sciences Division, Fred Hutchinson Cancer Center, Seattle, WA US; 4 Regeneron Pharmaceuticals, Tarrytown, NY US; 5 Division of Infectious Diseases, Massachusetts General Hospital, Harvard Medical School, Boston, MA US

**Keywords:** COVID-19, SARS-CoV-2, Post-exposure prophylaxis, Household transmission, Self-reported, Behavior

## Abstract

**Background:**

Following the outbreak of the COVID-19 pandemic, several clinical trials have evaluated postexposure prophylaxis (PEP) among close contacts of an index individual with a confirmed SARS-CoV-2 infection. Because index individuals do not directly inform the efficacy of prevention interventions, they are seldom enrolled in COVID-19 PEP studies. However, adjusting for prognostic covariates such as an index individual’s COVID-19 illness and risk behaviors can increase precision in PEP efficacy estimates, so approaches to accurately collecting this information about the index individual are needed. This analysis aimed to assess whether surveying household contacts captures the same information as surveying the index individual directly.

**Methods:**

REGN 2069/CoVPN 3502, a randomized controlled trial of COVID-19 PEP, enrolled household contacts of SARS-CoV-2 index individuals. CoVPN 3502-01 retrospectively enrolled and surveyed the index individuals. We compared responses to seven similar questions about the index individuals’ transmission cofactors that were asked in both studies. We estimated the percent concordance between index individuals and their household contacts on each question, with 50% concordance considered equivalent to random chance.

**Results:**

Concordance between index individuals and contacts was high on the most objective questions, approximately 97% (95% CI: 90–99%) for index individual age group and 96% (88–98%) for hospitalization. Concordance was moderate for symptoms, approximately 85% (75–91%). Concordance on questions related to the index individual’s behavior was only slightly better or no better than random: approximately 62% (51–72%) for whether they received COVID-19 treatment, 68% (57–77%) for sharing a bedroom, 70% (59–79%) for sharing a common room, and 49% (39–60%) for mask wearing at home. However, while contacts were surveyed within 96 h of the index individual testing positive for SARS-CoV-2, the median time to enrollment in CoVPN 3502-01 was 240 days, which may have caused recall bias in our results.

**Conclusions:**

Our results suggest a need to survey index individuals directly in order to accurately capture their transmission cofactors, rather than relying on their household contacts to report on their behavior. The lag in enrolling participants into CoVPN 3502-01 also highlights the importance of timely enrollment to minimize recall bias.

**Supplementary Information:**

The online version contains supplementary material available at 10.1186/s12889-024-18371-7.

## Introduction

Following the outbreak of the COVID-19 pandemic, several clinical trials have evaluated interventions for post-exposure prophylaxis (PEP) in the close contacts of an index individual with a confirmed SARS-CoV-2 infection, including hydroxychloroquine [1], monoclonal antibodies [2], antivirals [3, 4], and antiretrovirals [5]. Although studies primarily assessing COVID-19 transmission often enroll both the index individuals and their contacts [6, 7], to our knowledge no studies assessing COVID-19 PEP have enrolled index individuals because they do not directly inform the efficacy of prevention interventions. However, the severity of an index individual’s illness and their interaction with household members are important predictors of infection in their household contacts [8, 9], and adjusting for prognostic covariates such as these can increase the precision of PEP efficacy estimates [10].

Ideally, the best-quality information on an index individual’s COVID-19 illness and risk behaviors would be gained by direct observation (if possible) or by asking the person directly. However, in a PEP study in which index individuals are not enrolled, an alternative approach is to ask participants to report on the index individual in their household. Due to regulations including the International Council for Harmonisation (ICH) Guideline for Good Clinical Practice [11], with which the United States Food and Drug Administration (FDA) requires compliance, studies may not directly ask participants about an index individual who is not enrolled in the study; rather, investigators can ask participants about their own *observations* of the index individual (e.g., whether they *witnessed* symptoms in the index individual) under the hypothesis that this is sufficient to gain the same information as the index individual would report.

In this analysis, we consider two studies: a COVID-19 PEP study (REGN 2069/CoVPN 3502) [12] that enrolled household contacts of SARS-CoV-2 index individuals, and a later study (CoVPN 3502-01; the Index Individual study) [13] that retrospectively enrolled the index individuals. Because REGN 2069/CoVPN 3502 enrolled only household contacts, the study gathered information about the index individuals indirectly by surveying participants about the illness and risk behaviors (such as symptoms, mask wearing, and sharing rooms with other household members) of the index individual in their household.

We measure the concordance between household contacts’ responses about these transmission cofactors and the index individuals’ responses to similar questions that were asked of participants the Index Individual study. We aim to assess whether surveying household contacts about the index individuals can be a reliable substitute for surveying the index individuals directly. To our knowledge, the Index Individual study is the first to directly assess whether close contacts enrolled in a PEP trial can provide accurate proxy information about an index individual.

## Methods

REGN 2069/CoVPN 3502 (ClinicalTrials.gov identifier NCT04452318) [12] was a phase 3, randomized, double-blind, placebo-controlled study of a monoclonal antibody for COVID-19 PEP. The study enrolled asymptomatic household contacts aged 12 and over, who were randomized within 96 hours of an index individual’s COVID-19 diagnosis (defined as the time from diagnostic sample collection). In households with several contacts, all eligible contacts were enrolled in the study. The study was performed at 112 sites in the United States, Romania, and Moldova between July 2020 and February 2021. The primary endpoint was the percentage of participants with no evidence of previous SARS-CoV-2 infection at baseline who developed symptomatic RT-qPCR-confirmed SARS-CoV-2 infection within the 28-day follow-up period. As reported by O’Brien et al., the primary analysis found that the intervention significantly reduced the risk of SARS-CoV-2 infection among household contacts [2]. The present analysis of reported household transmission cofactors was not a prespecified secondary or exploratory objective of the study [14].

The Index Individual study (ClinicalTrials.gov identifier NCT05074719) [13] was a cross-sectional observational study that enrolled participants aged 10 and over with laboratory-confirmed SARS-CoV-2 whose household contacts were already enrolled in REGN 2069/CoVPN 3502 at sites in the United States. The study was performed remotely (online or by phone) by COVID-19 Prevention Trials Network (CoVPN) study staff. All enrolled participants provided written informed consent or assent with parental consent, and the protocol and other study materials were approved by the Western Institutional Review Board (IRB)–Copernicus Group (WCG) IRB. The study was performed between December 2020 and October 2021. The goal of the Index Individual study was to characterize index individuals to better understand the risk of transmission to household contacts enrolled in REGN 2069/CoVPN 3502; its primary objective was to build prediction models for SARS-CoV-2 acquisition among the household contacts by using the transmission cofactors recorded in the Index Individual study. The analysis presented here addresses a secondary objective of the study, which was to estimate concordance between the index individuals’ and their contacts’ recorded transmission cofactors.

The Index Individual study was originally designed to enroll participants within two weeks of their initial COVID-19 diagnosis (reflected in the Cohort A questionnaires); however, after the study began, delays in study activation and logistical hurdles in participant recruitment made it infeasible to enroll index individuals within this time frame, so the study questionnaires were redesigned to focus on retrospective data collection about participants’ past COVID-19 illness (reflected in the Cohort B questionnaires). Some households enrolled multiple individuals who self-reported SARS-CoV-2 infection concurrent with the initial infections in their household; for simplicity, we refer to them as “index individuals” throughout.

To assess whether surveying the household contacts in REGN 2069/CoVPN 3502 could be a substitute for surveying the index individuals directly, we compared responses to seven similar questions about the index individuals’ transmission cofactors that were asked in both studies: the index individual’s age group, whether they experienced COVID-19 symptoms, whether they were hospitalized for COVID-19, whether they received treatment for COVID-19, whether they shared a bedroom with other household members, whether they shared a common room with other household members, and whether anyone wore a mask at home (see Supplemental Table 1 for the exact wording of each question).

In REGN 2069/CoVPN 3502, participants were surveyed about the index individual at screening. The question about index individual age group was part of a survey administered to only one contact per household; otherwise, every other question considered here was answered by all contacts separately. In the Index Individual study, participants answered the questions in this analysis at either screening or baseline, with the exception of hospitalization: Cohort A participants reported whether they had been recently hospitalized for COVID-19 at the follow-up visits on Days 7 and 14. The questions about sharing rooms and mask wearing were part of a self-administered questionnaire in the Index Individual study; otherwise, all other questions in both studies were asked to participants by study staff.

For each question, concordance was defined as the proportion of contacts whose response matched that of at least one index individual in their household, to account for households that enrolled more than one self-reported index individual. Concordance estimates and 95% confidence intervals (CIs) for each question were computed using generalized estimating equations (GEE) with clustering by household and an exchangeable working correlation. Concordance of 50% is considered to be equivalent to random chance for all questions except index individual age group, in which concordance of 33% is equivalent to random chance due to there being 3 possible responses instead of 2.

We also performed several sensitivity analyses. In the first, concordance was estimated specifically in a subset of households with a household size of two, defined as households whose contact reported a household size of two and whose index individual reported one or two other members of the household. The aim of this analysis was to account for households that enrolled more than one index individual or contact: in households with more than one index individual, we cannot be sure to whom the contacts were referring in their responses, while in households with more than one contact, it is possible that one or more of the contacts later became infected and therefore could be mistakenly considered index individuals. We included households whose index individual reported either one or two other members of the household (rather than only one other member) to account for possible misunderstanding of the question by the index individual.

In the second sensitivity analysis, concordance was estimated among a subset of households with only Cohort B index individuals. The aim of this analysis was to account for potentially faulty responses from the Cohort A index individuals who were enrolled after the intended two-week time frame for the Cohort A questionnaire.

In the third sensitivity analysis, concordance was estimated among a subset of households whose index individuals were enrolled within 30 days of their contacts. This aim of this analysis was to account for potential recall bias from index individuals who reported on their illness more than a month after their first positive test.

## Results

REGN 2069/CoVPN 3502 randomized a total of 3,298 contacts from 2,523 households. Of the 2,523 potential index individuals, 114 were enrolled into the Index Individual study; this number is low because the Index Individual study initially only enrolled participants whose households had been enrolled at CoVPN study sites, which had fewer households than the Regeneron study sites. Study coordinators were able to identify the REGN 2069/CoVPN 3502 household IDs for 103 of these index individuals, matching them to a total of 79 households with 118 contacts enrolled in REGN 2069/CoVPN 3502. The 11 index individuals whose corresponding household IDs could not be determined are not included in this analysis; thus, the analysis comprises 103 index individuals in total. Thirty-three (33) households (42%) consisted of 2–3 people, and 35 (44%) consisted of 4–5 people. Seventeen (17) households (22%) enrolled more than one self-reported index individual. Age distribution was similar among index individuals and contacts: 16% of both index individuals and contacts were under 18 years of age. Among children under 18, the median age was 15 years old among index individuals and 16 years old among contacts. Of the 151 total index individual/contact pairs who shared a household, 89 (59%) were pairs of adults. The median time between household contact and index individual enrollment was 240 days; only 6 Cohort A index individuals were enrolled as intended within 2 weeks of their household contact(s) being enrolled. Table 1 details the demographics of the households and participants that were included in this analysis.

**Table 1:** Demographics

***Households***		
Total number of households	79	
Number of households of each size (as reported by index individual)^a^		
2–3 household members	33	(42%)
4–5 household members	35	(44%)
6–10 household members	13	(16%)
Number of households with each number of participants enrolled in Index Individual study		
1 index individual	62	(78%)
2 index individuals	11	(14%)
3 index individuals	5	(6%)
4 index individuals	1	(1%)
***Index individuals***		
Number of participants enrolled in Index Individual study	103	
Cohort A (questionnaires designed for data collection during acute phase of infection)	14	(14%)
Cohort B (questionnaires designed for retrospective data collection)	89	(86%)
Index individual age (years)	38	(20, 49)
Among index individuals under 18 years old	15	(14–16.25)
Number of index individuals enrolled per age group		
Child aged 10–17	16	(16%)
Adult aged 18–65	80	(78%)
Senior over 65	7	(7%)
***Household contacts***		
Number of household contacts enrolled in REGN 2069/CoVPN 3502	118	
SARS-CoV-2 negative at baseline	113	(96%)
SARS-CoV-2 positive at baseline	5	(4%)
Household contact age (years)	38	(21, 51.75)
Among household contacts under 18 years old	16	(13.5–16)
Number of household contacts enrolled per age group		
Child aged 12–17	19	(16%)
Adult aged 18–65	93	(79%)
Senior over 65	6	(5%)
***Index individual/contact pairs***		
Number of index individual/household contact pairs enrolled^b^	151	
Time from household contact to index individual enrollment (days)	240	(212.5, 276.5)
Cohort A index individuals	27.5	(6, 110.25)
Cohort B index individuals	241	(219, 282)
Number of index individual/household contact pairs enrolled in each age group		
Adult/adult	89	(59%)
Child/adult	22	(15%)
Adult/child	18	(12%)
Child/child	7	(5%)
Other	15	(10%)

Numbers reported are N (%) for count variables and median (IQR) for continuous variables. Counts not followed by a percent comprise the denominators for the counts that follow. Only participants in households with Index Individual study participants that were matched to REGN 2069/CoVPN 3502 participants are included in this table. ^a^In several households with more than one index individual, the index individuals reported different household sizes. ^b^In households with more than one index individual, each contact is a member of multiple pairs with different index individuals, resulting in more than 118 index individual/contact pairs

**Fig. 1:**
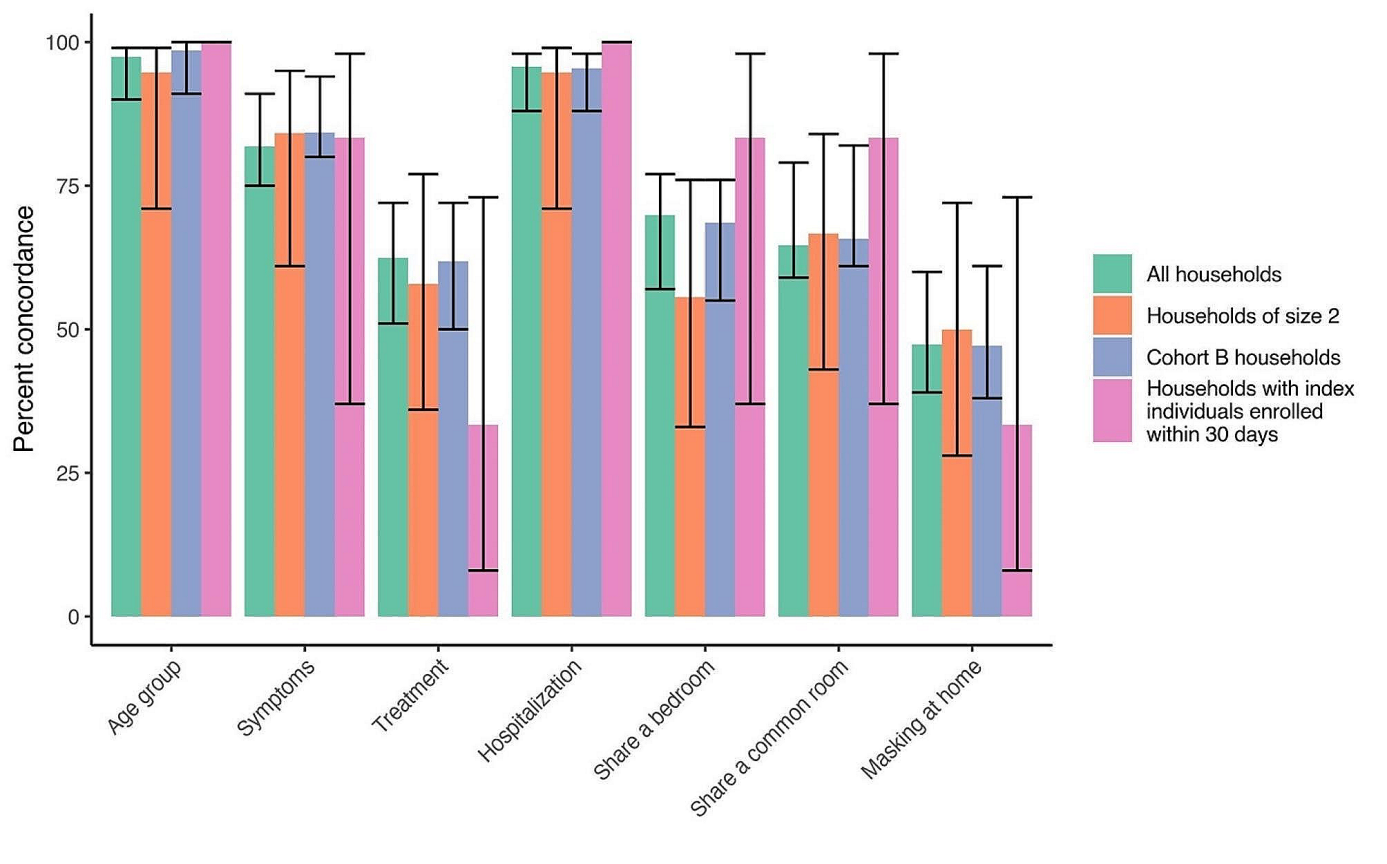
Concordance between household contacts and at least one index individual in their household calculated among all households (118 contacts, shown in green); among households of size 2 (19 contacts, shown in orange); among Cohort B households (110 contacts, shown in purple; Cohort B questionnaires were designed specifically for retrospective data collection in the Index Individual study); and among households whose index individuals were enrolled within 30 days of their household contacts (6 contacts, shown in pink). Black lines around the bars represent the 95% CIs for the concordance estimates

Figure 1 displays estimates of concordance. Among all 79 households (comprising 118 household contacts), concordance was approximately 97% (95% CI: 90–99%) for index individual age group, 85% (75–91%) for symptoms, 62% (51–72%) for treatment, 96% (88–98%) for hospitalization, 68% (57–77%) for shared bedroom, 70% (59–79%) for shared common room, and 49% (39–60%) for mask wearing at home.

Among the 19 households of size 2, concordance was approximately 95% (71–99%) for index individual age group, 84% (61–95%) for symptoms, 58% (36–77%) for treatment, 95% (71–99%) for hospitalization, 56% (33–76%) for shared bedroom, 67% (43–84%) for shared common room, and 50% (28–72%) for mask wearing at home.

Among the 71 households with only Cohort B index individuals (comprising 110 household contacts), concordance was approximately 99% (91–100%) for index individual age group, 89% (80–94%) for symptoms, 61% (50–72%) for treatment, 95% (88–98%) for hospitalization, 69% (55–76%) for shared bedroom, 73% (61–82%) for shared common room, and 49% (38–61%) for mask wearing at home.

Among the 6 households with index individuals enrolled 30 days or less after their contacts (comprising 6 household contacts), concordance was 100% for index individual age group, approximately 83% (37–98%) for symptoms, 33% (8–73%) for treatment, 100% for hospitalization, 83% (37–98%) for shared bedroom, 83% (37–98%) for shared common room, and 33% (8–73%) for mask wearing at home.

## Discussion

Although studies assessing household transmission commonly enroll both the index individual and their household contacts [6, 7], it is not common practice to enroll index individuals in PEP studies [1–5]. The Index Individual study supports the feasibility and importance of enrolling index individuals when studying cofactors of household transmission in the context of a PEP study, where an index individual’s illness and risk behaviors may provide more precision in transmission estimates. It is also an important example of a study that was successfully performed remotely, reducing costs and lowering COVID-19 exposure risk for both patients and clinicians [15].

Our results demonstrate that asking household contacts about their observations of an index individual does not always reliably generate the same information as would be provided by the index individual directly. Concordance was very high on index individual age group and hospitalization, which are the most objective questions considered here; however, concordance on index individual COVID-19 treatment, sharing a bedroom, and sharing a common room was only slightly better than random, and concordance on mask wearing at home was no better than random, as indicated by the lower limits of their 95% CIs being near or below 50%, respectively. The results in households of size two were similar, supporting our findings in households of all sizes, including households with more than one self-identified index individual or contact.

Previous COVID-19 transmission studies have shown that sharing a bedroom with the index individual significantly increases transmission risk, while self-reported mask wearing by contacts or the index individual significantly decreases transmission risk [8, 9]. In a PEP study, differences in household demographics, the severity of an index individual’s illness, and their interactions with household members can lead to very different risks of transmission within the household. Collecting data on transmission cofactors such as these can lead to more precise efficacy estimates for a PEP intervention by capturing the heterogeneity in transmission risk across households, which can explain some of the variability seen in the efficacy of the PEP intervention [10, 16]. Furthermore, because PEP studies prospectively follow individuals at high risk of becoming infected, they can be a valuable source of data on the effect of risk behaviors such as mask wearing on SARS-CoV-2 transmission, which is important for informing public health guidance for non-pharmacological interventions to decrease transmission, as highlighted in a recent review paper assessing such interventions [17]. Our results suggest that while surveying a close contact accurately captures the most objective information about an index individual, better methods to support observation are needed for capturing the index individual’s behaviors.

An important limitation to our results is the likely presence of recall bias in the index individuals’ answers to the questions about their COVID-19 illness: while REGN 2069/CoVPN 3502 enrolled and surveyed the household contacts within 96 hours of the index individual’s COVID-19 diagnosis, the Index Individual study enrolled and surveyed most index individuals many months later (Table 1) due to unforeseen delays in Index Individual study procedures. Recall bias is common in retrospective study designs and is known to increase as the length of the recall period increases, particularly for events that are routine or frequent [18]. Still, some studies suggest that recall ability is better for more significant events in one’s life [19]. Because the index individuals in this study were sick with COVID-19 during the first year of the global pandemic, when lockdowns were still in effect in many places and before vaccines were widely available, we expect that the events surrounding their illness would be especially memorable; however, we cannot rule out the possibility of recall bias affecting our results.

While concordance was very high for age and hospitalization—which we do not expect would be subject to recall bias—concordance was quite low for behavioral questions, which are more subjective. We expect that if the index individuals had been surveyed about their COVID-19 symptoms and treatment during the acute phase of their illness, their answers would be more accurate than those of their household members. Furthermore, because the questions about sharing rooms and mask wearing applied to the household as a whole and not the index individual specifically, we assume in this case that the household contacts provide more accurate information due to their enrollment during the index individual’s acute illness. However, neither the Index Individual study nor REGN 2069/CoVPN 3052 validated the responses to these questions, so we are unable to determine whether the index individual or contact’s response is “correct.” Thus, we had to limit our assessment to only whether the index individual and the contact reported the same information, not whether that information is accurate.

To address the potential recall bias, we performed a sensitivity analysis among households whose index individuals were surveyed within 30 days of their household contacts being surveyed. Our results for index individual age, hospitalization, and symptoms are consistent with the primary analysis. The results for the other questions were inconclusive: concordance on sharing a bedroom or common room was higher than the primary analysis, while concordance on treatment and masking at home was even lower than the primary analysis, though the 95% CIs are wide for all questions. Because of the small sample size (six contacts), we cannot draw any strong conclusions from these results, and a similar analysis should be repeated in the future with a larger sample size. Future studies should make a concerted effort to enroll individuals quickly when surveying them about their illness.

This analysis has several other limitations. First, due to the language in the Cohort A questionnaires (Supplemental Table 1), the eight index individuals in Cohort A who were enrolled more than two weeks after their initial diagnosis likely reported on the period of time after their infection had cleared. In a sensitivity analysis removing the Cohort A index individuals, the findings were in keeping with the primary results (Fig. 1). Second, because 22% of households enrolled more than one index individual (Table 1), we cannot be sure to whom their contacts were referring in their responses. Our definition of concordance (which considered agreement with *any* index individual in the household to be concordant) and our sensitivity analysis in households of size two addressed this question and demonstrated that the number of index individuals in the household did not change the results. Third, the behavioral questions in the Index Individual study were asked on a self-administered questionnaire instead of by study staff, which may have caused misinterpretation of these questions; on the other hand, social desirability bias may be less prevalent with self-administered questionnaires compared to study staff [20]. Future studies should utilize best practices for interviewing to minimize bias. Finally, although the questions in the Index Individual study were designed to collect the same information as the corresponding questions in REGN 2069/CoVPN 3502, the wording in many cases was either more or less precise, and therefore captured slightly different information, than in REGN 2069/CoVPN 3502 (Supplemental Table 1).

Direct observation of index individuals may better capture their transmission cofactors, particularly their behaviors such as sharing rooms with their household members. Future research should test improved methods to collect high-quality data on household transmission cofactors in the context of COVID-19 PEP studies.

### Electronic supplementary material

Below is the link to the electronic supplementary material.

Supplementary Material 1

## Data Availability

The datasets analyzed during the current study are available on reasonable request by contacting Laura Nakatsuka at Mass General Brigham (lnakatsuka@partners.org).

## Abbreviations

PEP: post-exposure prophylaxis
CI: confidence interval
CoVPN: COVID-19 Prevention Trials Network
FDA: United States Food and Drug Administration
GEE: generalized estimating equations
ICH: International Council for Harmonisation
IRB: institutional review board
