# Supplementary material for: Concordance between SARS-CoV-2 index individuals and their household contacts on index individual COVID-19 transmission cofactors: a comparison of self-reported and contact-reported information
Source: BMC Public Health. 2024 Apr 2;24:950. doi: 10.1186/s12889-024-18371-7 (PMC10986086; doi:10.1186/s12889-024-18371-7)
Supplement: Supplementary file 1 — Supplementary Material 1 [file 12889_2024_18371_MOESM1_ESM.docx]

| **Supplemental Table 1. Questions about the index individual asked in both studies** | |
| --- | --- |
| **Index Individual study** | **REGN 2069/CoVPN 3502** |
| Age (calculated as current date – date of birth) | Age group (senior above age 65/adult/child under age 18) |
| *Cohort A:* Are they experiencing symptoms of COVID? (yes/no)  *Cohort B:* Did the Index individual experience symptoms of COVID? (yes/no) | Have you witnessed or are you aware of any of the following symptoms of the suspected infected person or people in your home? (e.g. fever and/or cough, shortness of breath, chills, GI upset, loss of smell and/or taste) (yes/no) |
| *Cohort A:* Are you currently taking any medication for COVID-19? (yes/no)  *Cohort B:* Did you receive, or are you currently receiving antibodies and/or plasma treatments for COVID-19 through emergency use authorization? Did you take, or are you currently taking any other medication for COVID-19? (yes/no) | Are you aware of any treatment, investigational or approved, that the suspected infected person or people in your home may be receiving? (yes/no) |
| *Cohort A:* Over the last 7 days, were you hospitalized for COVID-19? (yes/no)  *Cohort B:* Have you been hospitalized for any period of time for COVID‑19/complications from COVID‑19? (yes/no) | Has any member of your home been hospitalized with COVID-19 in the past 4 weeks? (yes/no) |
| *Cohort A:* For the last week, what was the typical level of contact between the known or suspected infected person/s with COVID-19 in your home and persons without symptoms (or a negative COVID-19 test)? (check all that apply)  *Cohort B:* Within the week immediately following the time of symptom onset or COVID-19 diagnosis (whichever came first), what was the typical level of contact between the known or suspected infected person/s with COVID-19 in your home and persons without symptoms (or a negative COVID-19 test)? (check all that apply) | What is your level of contact to the known or suspected infected person or people in your home? (check all that apply) |
| Share a bedroom | Share a bedroom |
| Share a bathroom OR share a kitchen, OR share another common room, e.g. living room | Share a common room (kitchen, bathroom) |
| No one wears a mask in the home | No one wears a mask in home |
